# Predictors of Short-Term Mortality Following First Episode of Spontaneous Bacterial Peritonitis in Hospitalized Cirrhotic Patients

**DOI:** 10.7759/cureus.18999

**Published:** 2021-10-23

**Authors:** Abdel-Naser Elzouki, Abdelrahman Hamad, Hussam Almasri, Mohamed Ata, Anas Ashour, Muftah Othman, Ahmad Badi, Mehdi Errayes, Muhammad Zahid, Mohammed Danjuma

**Affiliations:** 1 Internal Medicine, Hamad Medical Corporation, Doha, QAT; 2 Internal Medicine, College of Medicine, Qatar University, Doha, QAT; 3 Internal Medicine, Weill Cornel Medical College, Doha, QAT; 4 Medicine, Hamad Medical Corporation, Doha, QAT

**Keywords:** infection, sepsis, mortality, cirrhosis, peritonitis, spontaneous bacterial peritonitis

## Abstract

Background and aims

Spontaneous bacterial peritonitis (SBP) is an important cause of morbidity and mortality in patients with cirrhosis. This study aimed to identify the factors impacting morbidity and short-term mortality in a cohort of patients with cirrhosis following an index episode of SBP.

Methods

In a retrospective study of hospitalized cirrhotic cohort, 333 patient records were reviewed. Demographic, clinical, and laboratory, as well as radiological characteristics of the patient population were analyzed on day 1 of admission. The diagnosis of cirrhosis was based on the combination of laboratory, clinical, and radiological features. The diagnosis of SBP was established by abdominal paracentesis in the presence of cellular, biochemical, and microbiological features consistent with SBP. All independent variables were analyzed to generate a predictive model of mortality by using the Cox proportional hazards regression analysis (adjusted for age and gender).

Results

A total of 61 cirrhotic patients with ascites and a first episode of SBP were identified. The overall mortality among hospitalized patients was 19.7% and was associated with longer length of stay (12.6 vs. 7.6 days; p=0.01). Patient cohorts with multiple antibiotic resistant bacteria as a cause of SBP had a significantly higher mortality compared to those with other bacterial phenotypes (p=0.03). Multivariate analyses showed that a model for end-stage liver disease (MELD) score (hazard ratio [HR]=1.29; 95% CI: 1.10 to 1.92; p=0.023), Child-Turcotte-Pugh score (HR=1.23; 95% CI: 1.05 to 1.82; p=0.027), and acute kidney injury (HR=2.09; 95% CI: 1.41 to 3.47; p=0.01) were the predictors of mortality from SBP.

Conclusion

SBP predicts in-hospital mortality in cirrhotic patients. In addition to multiple antibiotic resistant bacteria, thresholds of both hepatic and renal injury independently predict adverse outcomes.

## Introduction

Cirrhosis is considered a leading cause of morbidity and excess mortality, contributing to as much as over a million deaths annually. [1] This makes it the 11th commonest cause of death worldwide [1]. However, this figure probably underestimates the burden of cirrhosis as it is also associated with other pathologies such as hepatocellular carcinoma (HCC). Spontaneous bacterial peritonitis (SBP) represents one of the common causes of morbidity and excess mortality as a complication of ascites in patients with advanced liver cirrhosis [2]. It is an infection of the ascitic fluid, which almost always occurs in cirrhotic patients without a discernible, surgically treatable source within the abdomen. It is diagnosed by the presence of an elevated absolute polymorphonuclear (PMN) leukocyte count within the ascitic fluid of ≥250 cells/mm^3^, with or without positive culture, in the absence of secondary causes of peritonitis [2].

SPB has been associated with poor prognosis [3], and in spite of the progress made in its management and prevention, the rate of mortality among hospitalized patients ranges from 20% to 30% [4], with one-year mortality rate estimated at up to 50% in some studies [5]. Several factors have been reported to impact mortality in this cohort of patients. These include nosocomial infections, sepsis and septic shock, acute kidney injury (AKI) [6,7], and diagnosis as well as need for hospitalization [5], among others. The Child-Turcotte-Pugh (CTP) scores and the model for end-stage liver disease (MELD) are among the range of predictive algorithms that are suggested as predictors of mortality in hospitalized patients with cirrhosis and SBP [3,8].

The aim of this study was to examine the prognostic factors impacting short-term mortality in patients with cirrhosis following first episode of SBP. The predictive values of scores in routine clinical utility, i.e., CTP score and MELD score, were also evaluated.

## Materials and methods

Study population

In this observational study cohort, we reviewed 333 consecutive patients with cirrhosis who were admitted to Hamad General Hospital (i.e., a Weill Cornell affiliated tertiary hospital in Qatar). Patients with primary or secondary diagnosis of SBP were followed up until three days after diagnosis. Sociodemographic, laboratory, clinical, and radiological characteristics were analyzed on the first day of admission to the hospital.

Inclusion criteria

Cirrhosis was established based on the combination of laboratory, clinical, and radiological features. The diagnosis of SBP was confirmed by abdominal paracentesis in the presence of biochemical study consistent with SBP (i.e., presence of PMN leukocyte count ≥ 250 cells/mm^3^ in the ascitic fluid and positive microbiological culture) or with culture-negative neutrophilic ascites (i.e., PMN leukocyte count ≥ 250 /mm^3^ and negative culture). Short-term mortality was defined as 30-day in-hospital deaths. AKI was diagnosed utilizing the Acute Kidney Injury Network (AKIN) criteria [9], while sepsis and septic shock were defined consistent with the third international consensus definitions for Sepsis and Septic Shock (Sepsis-3) [10]. Hepatic encephalopathy was diagnosed according to the American Association for Study of Liver Disease (AASLD) and European Association for Study of Liver (EASL) guidelines [11], and multiple antibiotic resistant bacteria was defined as recently reported by the British Infection Association [12]. CTP and MELD scores were estimated following the diagnosis of first SBP episode. CTP score denotes the extent of liver failure on a numerical scale of 5-15 based on the results of laboratory and clinical variables [13]. MELD score is an extensively validated mathematical algorithm for the prediction of mortality of patients with liver disease. It is routinely used for the evaluation of cirrhotic patients requiring liver transplantation [8].

Exclusion criteria

Cirrhotic patients with ascites were excluded from the study if (a) ascites fluid PMN leukocyte count was less than 250 cells/mm^3^; (b) there was a presence of acute variceal hemorrhage; (c) there was a presence of advanced malignancy including HCC; (d) there was clinical suspicion of secondary peritonitis suggested by one of the following: a positive microbial culture, glucose levels < 50 mg/dL, or supportive radiological imagine signs; and/or (e) there was a prior episode of SBP.

Statistical analysis

We described continuous variables as mean ± standard deviation (SD), and differences between variables were compared utilizing Student's t-test as appropriate. Categorical covariates were presented as number (%) analyzed using a chi-square test. All gender- and age-adjusted variables were analyzed to generate Cox proportional hazards regression predictive multivariate models of mortality following SBP. Variables with p-values < 0.05 were considered significant. All analyses were carried out using SPSS software Version 22.0 (IBM, Armonk, NY, USA).

Ethical considerations

All study documentations including its protocol were approved by the Medical Research Centre (MRC) of Hamad Medical Corporation, Qatar (Approval: MRC number 13302/13).

## Results

Patient characteristics and outcomes

A cohort comprised 61 cirrhotic patients with ascites and first episode of SBP that satisfied all the pre-specified inclusion criteria. The demographic/clinical parameters of the study population are shown in Table 1. There was a high proportion of male patients’ cohort (85.2% [52/61]), with a mean age of 49.1±12.4 years. Unadjusted in-hospital mortality rate was 19.7% (n=12). Age- and gender-adjusted mortality as well as etiology of liver cirrhosis remained the same between SBP survivors and SBP deaths (Table 1).

**Table 1:** Clinical characteristics of hospitalized cirrhotic patients with SBP classified according to the short-term mortality outcome (N=61) MELD, model for end-stage of liver disease; NAFLD, nonalcoholic fatty liver disease; NS, not significant; SBP, spontaneous bacterial peritonitis

Characteristics	SBP survivors (n= 49), N (%)	SBP deaths (n= 12), N (%)	p-Value
Demographic data			
Age: mean±SD (year)	48.8±11.7	49.8±13.5	NS
Male (%)	42 (85.7)	10 (83.3)	NS
Etiology of cirrhosis			
Hepatitis B infection	7 (14.3)	2 (16.7)	NS
Hepatitis C infection	21 (42.8)	5 (41.7)	NS
Ethanol	18 (36.7)	4 (33.3)	NS
Cryptogenic/NAFLD	3 (6.1)	1 (8.3)	NS
Clinical data			
Acute kidney injury	11 (22.4)	6 (50)	0.01
Hepatic encephalopathy	12 (24.5)	7 (58.3)	0.01
severe sepsis/septic shock	8 (16.3)	6 (50)	0.001
Diabetes mellitus	26 (53)	6 (50)	NS
Positive ascitic fluid culture	10 (20.4)	3 (25)	NS
Multiple antibiotic resistant bacteria	1 (4.9)	2 (16.6)	0.03
Scores (mean±SD)			
Child-Turcotte-Pugh (B/C)	10.6 ± 0.9	11.1 ± 1.03	0.027
MELD score (cut-off value: 17)	24.9 ± 7.3	31.3 ± 6.1	0.023

Multi-morbidities were proportionately higher among those who died compared to survivors, including AKI (50% vs. 22.4%; p=0.01), hepatic encephalopathy (58.3% vs. 24.5%; p=0.01), and sepsis (50% vs. 16.3%; p=0.001). The exception was patients with type 2 diabetes mellitus and positive ascitic fluid culture, whose point estimate was similar among both SBP death and SBP survivor groups (50% vs. 53%, p=nonsignificant [NS] and 25% vs. 20.4%, p=NS; respectively). In-hospital mortality was proportionately higher in patients with multiple antibiotic resistant bacteria compared to those with other bacterial isolates (16.6% vs. 4.9%, p=0.03) (Table 1). A longer length of stay in hospital was associated with increased in-hospital mortality (12.6 days vs. 7.6 days, p=0.01), leading to significantly higher hospital resource utilization and cost.

Predictors of in-hospital mortality

Table 2 shows the predictive model of SBP mortality of all independent variables using Cox proportional hazards regression model analysis. MELD score (hazard ratio [HR]: 1.29; 95% confidence interval [CI]: 1.10 to 1.92; p=0.023); CTP (B/C) score (HR=1.23; 95% CI: 1.05 to 1.82; p=0.027), and AKI (HR=2.09; 95% CI: 1.41 to 3.47; p=0.01) were significantly predictive of in-hospital mortality. The MELD value of 17 was the optimal cut-off point for predicting a worse prognosis and poor outcome.

**Table 2:** Multivariate Cox regression analysis of risk factors for spontaneous bacterial peritonitis related mortality MELD, model for end-stage of liver disease

Independent variables	Hazard ratio	95.0% confidence interval		p-Value
		Lower	Upper	
Acute kidney injury	2.09	1.41	3.47	0.01
Child-Turcotte-Pugh score	1.23	1.05	1.82	0.027
MELD score	1.29	1.10	1.92	0.023

## Discussion

Bacterial infections represent a common and serious complication in cirrhotic patients. This often accounts for its significant morbidity and excess mortality, as well as one of the most common causes for repeated hospital admissions in this cohort of patients [14]. Development of infection is frequently associated with AKI [7], acute-on-chronic liver failure [15], and significant increase in mortality. A meta-analysis evaluating 178 studies and 11,987 patients with cirrhosis demonstrated that bacterial infections increase overall mortality by four-fold [16].

SBP is among the most common types of infections in patients with liver cirrhosis [5]. It is estimated to represent around 25% to 31% of all infections in these patients and a prevalence ranging from 10% to 30% in inpatient cirrhotic cohorts with ascites [5]. The occurrence of first episode of SBP is considered an inflection point in the natural history of cirrhosis and ascites, denoting further decompensation and profound impact on survival (even after resolution of SBP) [3]. The 30-day in-hospital mortality of SBP is variable but has been reported to be anywhere between 18% [17] and up to 31.9% [16]. Long-term three-year mortality of SBP has been estimated to be around 66.5% in one study [18], with a 2.5-fold increase in mortality compared to those without ascites. In our study, the unadjusted 30-day in-hospital mortality was 19.7%, which is in the lower spectrum of that reported from recent systematic reviews. We attribute this lower mortality rate in our report to be possibly due to exclusion of patients with variceal bleeding and advanced malignancy including HCC; this contrasts with most of the studies in this area (which included them in their cohorts). The relatively lower mean age of patients included in our study could also be a contributing factor for lower mortality rate in our cohort. Determining the prognosis of SBP would be essential for formulating a proper management plan, as well as selection of robust antimicrobial coverage, and setting up appropriate follow-up schedules. Several prognostic factors have been suggested as predictors of mortality in patients with SBP. These include serum urea, white blood cell count, and CTP and MELD scores [19]. Additionally, Tsung et al. reported that higher serum total bilirubin levels, a prolonged prothrombin time, low ascitic glucose levels, and the presence of HCC were associated with a proportionately higher mortality rate in cirrhotic patients with SBP [20].

In our study, CTP score, MELD score, and AKI were associated with excess in-hospital mortality. Development of hepatic encephalopathy and septic shock were also associated with increased short-term mortality in our study. Impairment of liver function, as suggested by the MELD score, was reported as a good predictor of mortality in SBP in several studies throughout the literature [3]. Several MELD values, such as 16.5 [3], 20.5 [21], and 22 [22], have been suggested as optimal cut-off points for predicting a worse prognosis in these cohorts of patients. In our study, a MELD value of 17 was the optimal cut-off point associated with poor outcome. This is well within the range of 16-22 reported in previous studies [3,22].

Patients with SBP have a higher likelihood of developing an AKI, and this morbidity is associated with an approximately six-fold increase in the risk of mortality in cirrhotic patients with SBP [23]. In a study from a large nationwide retrospective cohort in the United States, 70.3% of all patients who died with SBP had an AKI [24]. In a systematic review of 18 studies aimed at identifying the most consistent predictors of excess mortality in patients with SBP on a background of cirrhosis, renal dysfunction was found to be the main predictor of adverse outcome for cirrhotic patients with SBP, followed by the MELD score [7]. This is consistent with the finding from our study, which showed a significant increase in excess mortality in patients with AKI compared to patients with normal kidney function.

Our study showed a significantly higher mortality rate in patient cohort with multi-drug resistant (MDR) bacteria than in those with other bacterial isolates. In general, development of MDR bacterial morbidity in patients with cirrhosis is a poor prognostic marker and has been shown to be an independent risk factor for excess mortality [25]. In recent years, the epidemiology of bacterial infections in cirrhosis has witnessed a rising prevalence of MDR, leading to higher incidence of treatment failure, with subsequent development of septic shock, deterioration of liver function, and eventually increased mortality [26]. Identical findings were reported in a prospective study in Germany, which also demonstrated a high treatment failure rate associated with the first-line antibiotic regimens in the treatment of SBP [27]. In one retrospective study of 246 episodes of culture-positive SBP, third-generation cephalosporin resistance occurred in 21.5% [28]. This rate of cephalosporin resistance was also reported to be as high as 45% in a study conducted in the United States [29]. Patients with nosocomial SBP caused by MDR bacteria had higher a 30-day mortality compared with common bacteria [25]. In fact, MDR bacteria were isolated in up to 22% of SBP cases [30], and they had a frequency of isolation in cohorts with healthcare-associated infections, patients stabilized on long-term prophylaxis with quinolones-based antibiotics, and those with higher MELD score [31]. Therefore, it is crucial that evaluation of individual risk factors for acquiring MDR organism infection, and the distinction between community-acquired, healthcare-associated or nosocomial infections, should guide the choice of empirical antibiotic therapy. Our study is limited by the usual liabilities associated with retrospective data review (including missing data and proper adjudication of clinical outcomes).

## Conclusions

We found that a first episode of SBP predicts high morbidity and short-term excess mortality in patients with cirrhosis. The most consistent prognostic factors were the presence of renal impairment, infections with MDR bacteria, and advanced liver disease (as evidenced by higher CTP and MELD scores). Our study will assist healthcare professionals in the risk stratification of these cohorts of patients, especially channeling therapeutic interventions to prevent and delay first episodes of SBP.
